# Development and validation of the coffee task: a novel functional assessment for prosthetic grip selection

**DOI:** 10.1186/s12984-024-01307-y

**Published:** 2024-02-08

**Authors:** Christina Lee, Alex K. Vaskov, Alicia J. Davis, Jordan M. Kartes, Deanna H. Gates

**Affiliations:** 1 Department of Biomedical Engineering, University of Michigan, Ann Arbor, MI USA; 2 John A. Paulson School of Engineering and Applied Sciences, Harvard University, Boston, MA USA; 3 Section of Plastic Surgery, University of Michigan, Ann Arbor, MI USA; 4 Orthotics and Prosthetics Center, University of Michigan, Ann Arbor, MI USA; 5 School of Kinesiology, University of Michigan, Ann Arbor, MI USA; 6 Department of Robotics, University of Michigan, Ann Arbor, MI USA

**Keywords:** Prostheses and implants, Upper Extremity, Rehabilitation, Functional Assessment

## Abstract

**Background:**

Lack of standardized assessments that explicitly quantify performance during prosthetic grip selection poses difficulty determining whether efforts to improve the design of multi-grip hands and their control approaches are successful. In this study, we developed and validated a novel assessment of multi-grip prosthetic performance: The Coffee Task.

**Methods:**

Individuals without limb loss completed the Box and Block Test and two versions of the Coffee Task – Continuous and Segmented - with a myoelectric prosthetic emulator. On different days, participants selected prosthetic grips using pattern recognition and trigger control. Outcomes of the Continuous and Segmented Coffee Task were completion time and number of errors, respectively. Two independent raters assessed outcomes of the Coffee Task using video recordings to determine inter-rater reliability. Known-group validity was assessed by comparing outcomes with the emulator to those with an intact limb. Convergent validity was assessed through the correlation of the Coffee Task outcomes and those of the Box and Blocks Test. Responsiveness to changes with practice and control approach were assessed using the standardized response mean (SRM).

**Results:**

Inter-rater reliability was high for both versions of the Coffee Task (Intra-class coefficient > 0.981). Coffee Task outcomes were moderately correlated with the Box and Blocks outcomes (|r| ≥ 0.412, *p* ≤ 0.007). Participants completed the Coffee Task faster with their intact limb than with the emulator (*p* < 0.001). Both versions of the Coffee Task were responsive to changes with training (SRM ≥ 0.81) but not control approach (SRM ≤ 0.12).

**Conclusions:**

The Coffee Task is reliable, has good known-group and convergent validity, and is responsive to changes due to practice. Future work should assess whether the Coffee Task is feasible and reliable for people with upper limb loss who use multi-grip prostheses.

**Supplementary Information:**

The online version contains supplementary material available at 10.1186/s12984-024-01307-y.

## Background

While the use of an upper limb prosthesis is positively correlated with increased health-related quality of life [1], employment rates [1–3], and ability to accomplish activities of daily living [4], over a quarter of people with upper limb loss choose not to wear one, citing a lack of perceived comfort and function [5, 6]. Numerous approaches have been developed to address the lack of prosthetic function, including enabling multiple grip functions to facilitate fine motor skills. These features have been requested by prosthesis users, and they self-report that use of multiple articulating fingers facilitates easier and more reliable grasping of objects compared to single-grip hands [7–9]. Multi-grip hands use a variety of different control approaches to enable the user to change their grip including: trigger control, with which the user selects grips using electromyography (EMG) triggers; gesture control, with which the user selects grips through movement of the limb recorded via sensors in the prosthesis [10]; and EMG pattern recognition [10, 11]. Each approach has limitations and is reported to be both challenging to learn and cognitively demanding [12, 13].

While there has been significant investment in improving both the design of hands and their control approaches, it has been difficult to determine if these efforts are successful due to a lack of common assessment tool that enables objective comparison of different multi-grip prostheses and their controllers. Studies that investigated performance during grip selection with different control approaches have used assessments that either do not quantify grip transition performance [14–18] or are created specifically for that study and thus difficult to compare across approaches [19, 20]. Existing outcome measures to assess prosthetic dexterity include a mix of rater-dependent evaluations of function, patient-reported outcome measures, and objective functional tests [21]. Rater-dependent evaluations, such as the Assessment of Capacity for Myoelectric Control (ACMC) and Activities Measure for Upper Limb Amputees (AMULA) have the advantage of measuring function during daily tasks, but they can be difficult to administer as they rely on an observer with extensive training to rate performance [22, 23]. Additionally, the ACMC is validated for users of myoelectric prostheses, but not necessarily multi-grip prostheses, and thus does not explicitly assess the ability to select appropriate grips [9]. Other existing functional assessments, such as the Southampton Hand Assessment Procedure (SHAP), use task completion time to evaluate hand function using multiple grips during unilateral movements that require a single grip at a time [24, 25]. Grip switching performance is not evaluated in any measurable portion of the task. Therefore, the assessment indirectly quantifies prosthesis users’ ability to select the optimal grip to complete the given task, as the use of suboptimal grip will result in a longer completion time. Previous studies have modified the SHAP to require the person to move a sequence of objects that each require different grips [9, 19]. However, these modifications are inconsistent between studies and have not been validated.

To address the limitations of current validated outcome measures, we designed the Coffee Task to assess prosthetic grip selection performance. This assessment was designed to be easy to administer and score in clinical and research environments, inexpensive to implement, and produce easily interpretable outcomes. To determine if this assessment was feasible and valid for assessing grip switching performance, we evaluated its inter-rater reliability, construct validity, known-group validity, and responsiveness to change with practice and control approach.

## Methods

### Development of the functional task

We developed an assessment with the requirements that it (1) includes grip selection as part of the assessment, (2) represents an activity of daily living, (3) uses minimal and inexpensive equipment, (4) be easy to implement for clinical or research use, and (5) be easy to score and interpret outcomes. From these criteria, we developed the Coffee Task, during which the individual brews a cup of coffee in a pod-style coffee maker.

**Fig. 1:**
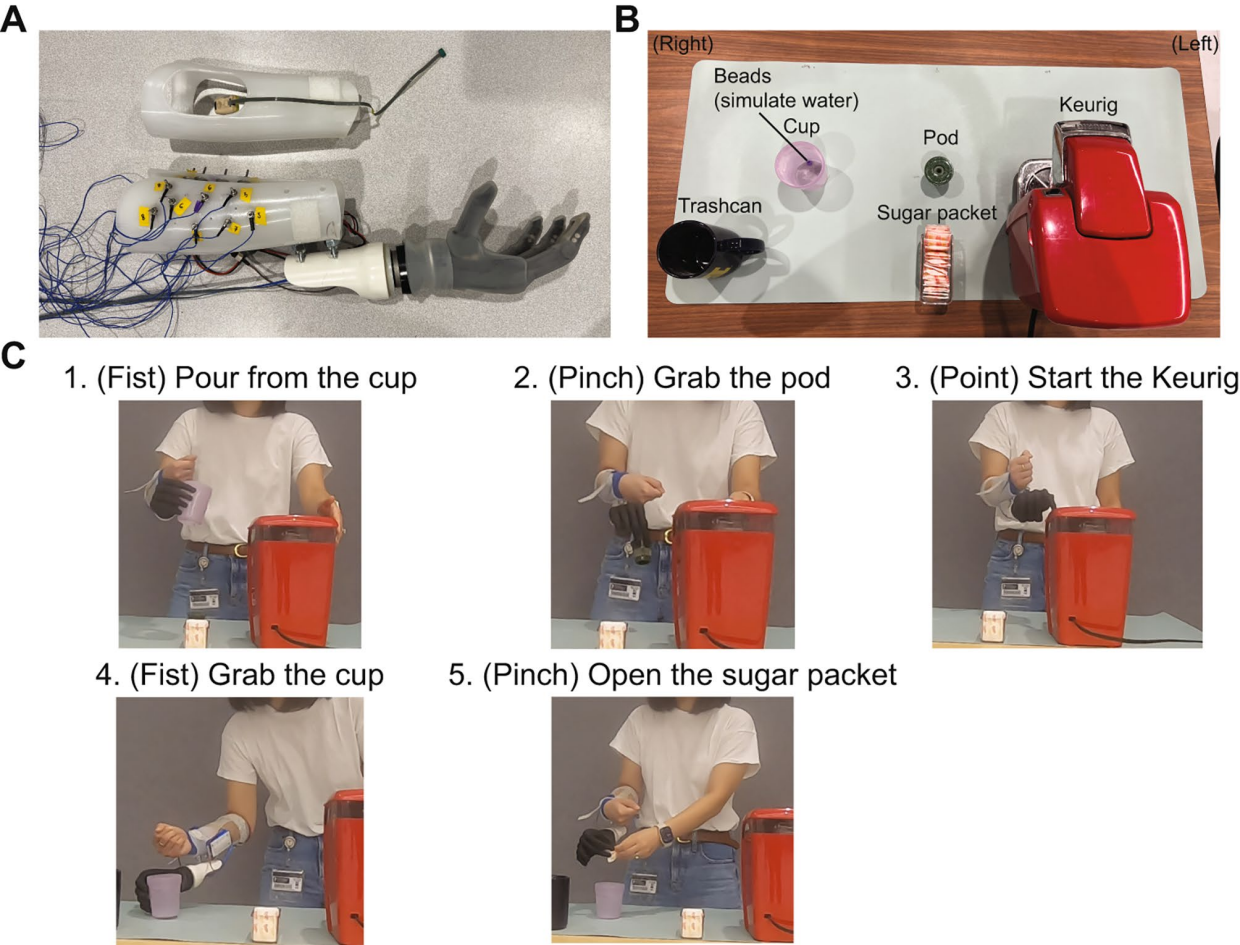
(**A**) Prosthetic emulator set-up for trigger control with dual-site electrodes (top) and pattern recognition with eight pairs of dome-shaped electrodes (bottom). The prosthesis was attached to the emulator socket such that it was below the right anatomical limb when the forearm was in a neutral position in front of the body. There was a 2-cm lateral gap between the emulator socket and the prosthesis using two bolts to allow movement of the anatomical right hand, which was necessary to control the prosthesis. (**B**) Set up of the Coffee Task. Participants interacted with the task objects that were placed in their designated area on the placemat. The height of the table with the placemat was aligned with their greater trochanter. (**C**) Individual segments of the Coffee Task. Participants were asked to complete the task both continuously and in segments

The Coffee Task is completed in the following sequence (Fig. 1): (1) open the water reservoir of a pod-style coffee maker (Keurig mini (Reading, MA)) with the contralateral intact limb and use the fist (power) grip with the prosthesis to grab the coffee cup and pour water (simulated with two small beads) into the reservoir, (2) open the pod holder with the intact limb and use the tripod pinch grip with the prosthesis to grasp the coffee pod and place into the pod holder, (3) use the point grip with the prosthesis to push the start button, (4) use the fist grip with the prosthesis to move the coffee cup from the coffee maker to the table, and (5) use the intact limb and the pinch grip with the prosthesis to open a sugar packet and pour into the cup. The prosthetic grips were chosen as they are helpful for completing different segments of the task and are among the most common of the 11 grip and hand postures reportedly used by prosthesis users in daily life [9].

We developed two versions of this assessment: The Segmented and the Continuous Coffee Task. In the Segmented Coffee Task, the individual completes each of the five segments individually, with up to five attempts to complete all parts of the segment accurately including correct grip selection. If the participant made an error during a particular segment, the assessment paused and the rater reset any materials used in that segment so the participant could make an additional attempt. The primary outcome of the Segmented Coffee Task is the number of errors, with a maximum total error of 25. In the Continuous Coffee Task, the individual completes all segments of the task continuously without pausing, with the primary goal of completing the task as quickly as possible. Although the use of inaccurate grips is not explicitly penalized, the individuals are instructed to complete as many parts of the task accurately as possible. The individuals should also not use their intact limb to complete parts of the task that are meant to be completed with their prosthesis. The primary outcome of the Continuous Coffee Task is the completion time, with a maximum time of 150 s.

We selected upper bounds of each version of the task based on pilot testing with three individuals. These values were selected to reduce the fatigue and frustration that were expressed when the participant did not have good control over the prosthesis. These values also enabled us to capture a broad range of skills in these completely novice pilot participants without substantial ceiling effect.

### Participants

Based on power analysis, 10 participants would yield 80% power to detect intra-class coefficient (ICC) of 0.75. Eleven healthy adults (age 28.4$\pm$4.4 years, 5 males) without a history of neurological or musculoskeletal conditions affecting their movement participated after confirming their right hand dominance based on the modified Oldfield’s handedness questionnaire [26]. All participants provided their written informed consent to participate in this institutionally approved study.

### Experimental protocol

**Fig. 2:**
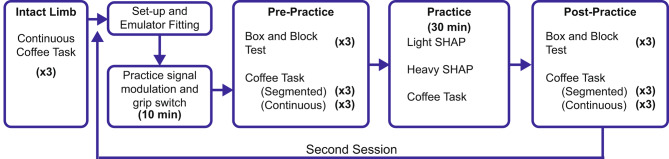
Experimental Protocol. Participants completed two sessions, at least one-week apart, each with a different grip-selection control approach (i.e., trigger control, pattern recognition), in random order

The participants completed two experiment sessions, spaced at least one week apart (Fig. 2) where they were assigned to use one of two control approaches in the first session, followed by the second approach in the next session. In the first session only, participants completed three trials of the Continuous Coffee Task without the prosthetic emulator. They did not complete the Segmented Coffee Task with the intact limb since individuals without amputation do not use discrete grips. Participants were then fitted with a prosthetic emulator with an extra-small or a small right-hand i-Limb Quantum (Ossur, Reyjavik, Iceland) with an average weight of 1.04 lbs and taught how to use a given control approach (see 2.4 Control Approaches). Once they were successfully able to switch to each grip (~ 20 min), participants completed the Box and Block Test (BBT) of manual dexterity [27] and both versions of the Coffee Task (‘Pre’). The participants completed the Segmented Coffee Task first, followed by the Continuous Coffee Task. Participants then had 30 min to practice grasp transitions while manipulating objects. This period was divided into three 10-minute sections, each of which consisted of seven minutes of practice and three minutes of rest. The sections were (1) manipulating light objects from the SHAP [24], (2) manipulating heavy SHAP objects, and (3) practicing the Coffee Task. These practice sessions were unstructured and participant-driven such that they could incorporate the available objects however they saw appropriate, as long as they were practicing with the goal of improving their grip transitions. All objects were placed on a height-adjustable table that was aligned with the participant’s greater trochanter to accommodate for the lack of prosthetic wrist motion and the placement of the prosthesis inferior to the arm (Fig. 1). After training, participants repeated the BBT and both versions of the Coffee Task (‘Post’). Based on pilot testing, all functional assessments were completed three times per condition to minimize fatigue. All assessments were video recorded from three different views – frontal, sagittal, and diagonally behind the left hand.

As the task was designed to assess performance during grip switching, it required participants to complete multiple steps in sequence. To determine if the task was too challenging, we asked the participants to complete the NASA-Task Load Index of perceived workload [28] regarding both versions of Coffee Task during Post. Participants weighed the relative importance of six aspects of workload (i.e., mental demand, physical demand, temporal demand, performance, effort, frustration) as well as the perceived workload in each aspect on a 0-100 scale. The calculated weighted workload ranged from 0 (least workload) to 100 (highest workload) [29].

### Control approaches

At the start of each session, participants were trained to switch prosthetic grips using either trigger control or pattern recognition. Trigger control used two bipolar surface EMG electrodes (Ottobock, Duderstadt, Germany) placed on an agonist-antagonist muscle pair. Participants were taught to use three distinct muscle triggers: hold open during which the participants held an isolated extensor signal for two seconds, double impulse during which the participants applied two bursts of extensor signal, and triple impulse during which the participants applied three bursts of extensor signal. The grip-trigger pairing was randomly assigned to each participant. Pattern recognition used eight pairs of EMG electrodes (College Park, Warren, MI) to establish participant-specific classifiers using linear discriminant analysis (LDA), as this approach is common in commercial systems. Classifiers were calibrated for each participant using the mean absolute value of rectified and 100–500 Hz band-pass filtered EMG to predict no movement, the three grips, or hand open [30]. Channels with visually high baseline noise during calibration were excluded after verifying that their inclusion reduced predicted classification accuracy (average 1, range 0–3 channels removed). Quality of calibration was visually confirmed with a real-time virtual environment grip selection task [31] before starting the standardized assessments. Prior to any assessment, the participants demonstrated that they could control the grip aperture and transition to all grips used in the Coffee Task at least once during training.

### Data analysis

A rubric to classify error types during the Segmented Coffee Task was developed after consulting with a certified prosthetist/orthotist (AD). Two individuals (CL and JK) with background upper limb prosthesis research discussed the interpretation of different error types with a representative participant data. Following this discussion, the raters independently quantified the number of errors from the Segmented Coffee Task by type (Additional File 1 & 2) and the completion time from the Continuous Coffee Task using video recordings. We assessed *inter-rater reliability* between the two raters’ assessment of transition errors using an intra-class correlation (ICC_2,1_) for a single measure in absolute agreement. ICCs were considered valid when significant F-tests (*p* < 0.05) indicated sufficient heterogeneity [32]. ICCs > 0.90 indicate reasonable reliability for clinical measures, ICCs > 0.75 indicate good reliability, and ICCs < 0.75 indicate poor to moderate reliability [32]. All subsequent analyses used data from a single rater.

We first explored learning effects across the three trials of the Coffee Task using a one-way analysis of variance (ANOVA) with trial number as a fixed factor. As there was no significant effect of trial number (*p* > 0.413; Additional File 3), we averaged across all trials within a block (Pre/Post). We performed a distributional analysis of the average outcomes to determine whether the distribution of outcomes was skewed [33]. We considered floor or ceiling effects to be present if more than 15% of the outcomes were either the minimum or the maximum possible score.

We examined the *known-group validity* of the Coffee Task by comparing the completion time between all trials completed with the prosthetic emulator with trigger control during Pre and those completed without the emulator using a 2-tailed paired t-test. *Convergent validity* was assessed using Pearson’s correlations between the outcomes of the two versions of the Coffee Task and the BBT. Correlations were considered weak (0 < *r* < 0.3), moderate (0.3 < *r* < 0.6), or strong (*r* > 0.7) [34].

Finally, we assessed *responsiveness* of the Coffee Task to practice (Pre/Post) and control approaches using the standardized response mean (SRM) and effect size. The SRM was calculated as the mean of the difference in outcomes between conditions divided by the standard deviation of the difference. A higher SRM indicates greater sensitivity to change with an SRM less than 0.5 considered to be insensitive to change [35]. Effect size (Cohen’s d) was the difference between the average of two conditions divided by the pooled standard deviation of the two conditions. An effect size > 0.8 was considered large, 0.5 to 0.8 was moderate, and 0.2 to 0.5 was small [36]. All statistical analyses were performed using SPSS (version 28, IBM Corp, Chicago, IL) with a significance level of α = 0.05.

## Results

### Summary of included data

Data from two trials (Participant 6, Pre; Participant 9, Post) with pattern recognition were omitted due to technical difficulties. In the Segmented Coffee Task, there were seven trials, or 5.5% of all trials collected, where fewer than five unsuccessful attempts were made in a given segment before the participant proceeded to the next segment. In these cases, the raters added the number of errors to record five, or the maximum number of errors possible, but did not record error type (< 2% of all errors).

### Inter-rater reliability

ICCs for interrater reliability were 0.991 for the Continuous Coffee Task and 0.981 for the Segmented Coffee Task. Of note, the ICC for the Segmented Coffee Task reflects only the number of errors, not the error type, which occasionally differed between raters (Additional File 4). For pattern recognition, the most common type of error observed was a command classification error where the prosthetic hand transitioned to the incorrect grasp. For trigger control, the most common error was a ‘no response’ error where the trigger was applied, but the transition did not occur.

### Distribution of data

**Fig. 3:**
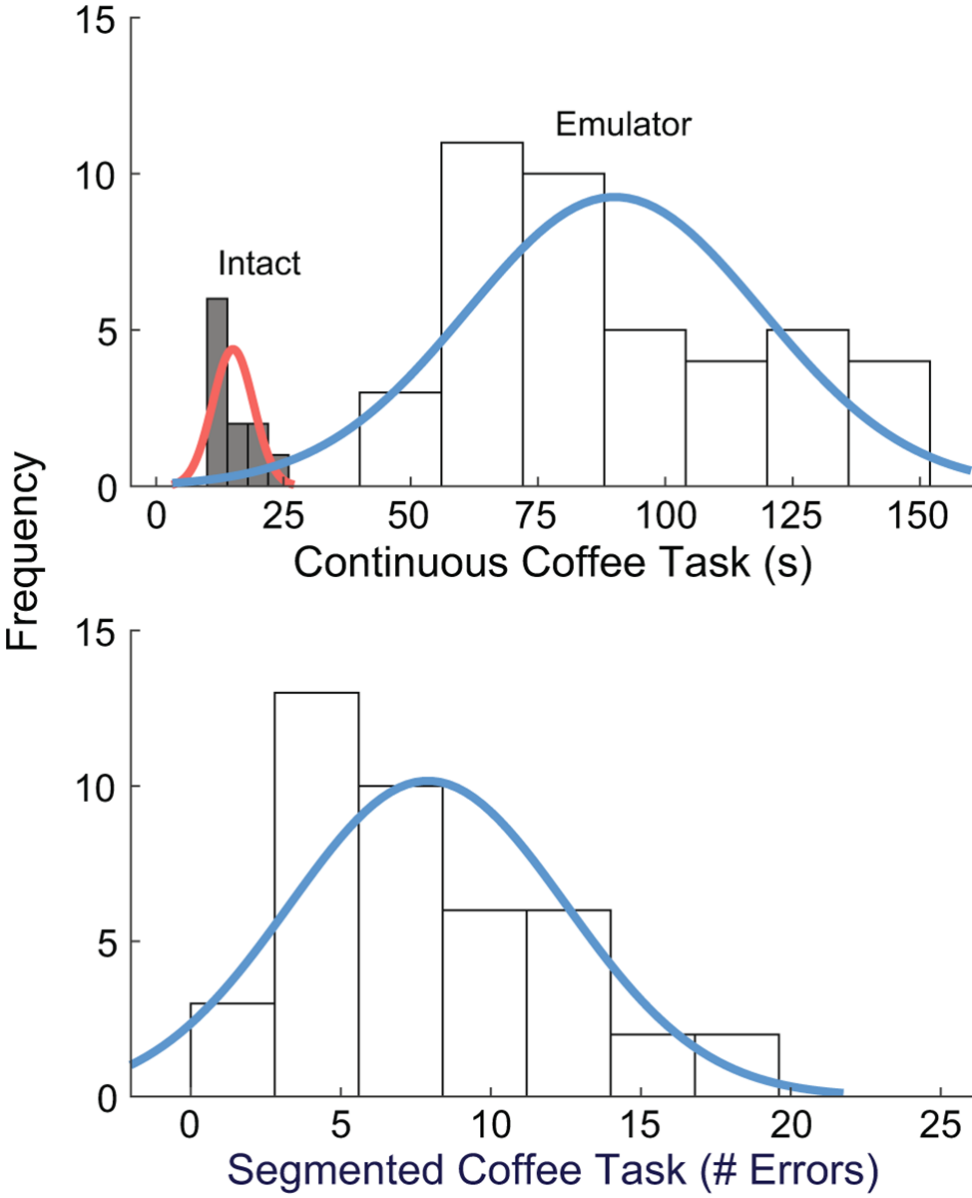
Histogram of average outcomes of the Continuous (top) and Segmented (bottom) Coffee Task for both control approaches Pre and Post-training. Data for movements with an intact limb (i.e. no emulator) are shown for the Continuous Coffee Task only as segmental errors are not possible to quantify with intact limbs. Neither version of the Coffee Task demonstrated ceiling or floor effects. Both Coffee Task outcomes were skewed toward lower values

Participants made an average of 7.9 ± 4.6 errors during the Segmented Coffee Task and took an average of 89.6 ± 28.4 s to complete the Continuous Coffee Task (Fig. 3). Distributions were skewed toward lower values (skewness = 0.56 for completion time and 0.55 for number of errors). Neither distribution for the two versions of the task indicated ceiling or floor effects (0% and 4.8% of errors and time had the maximum or minimum possible score, respectively).

### Known-group validity and convergent validity

**Fig. 4:**
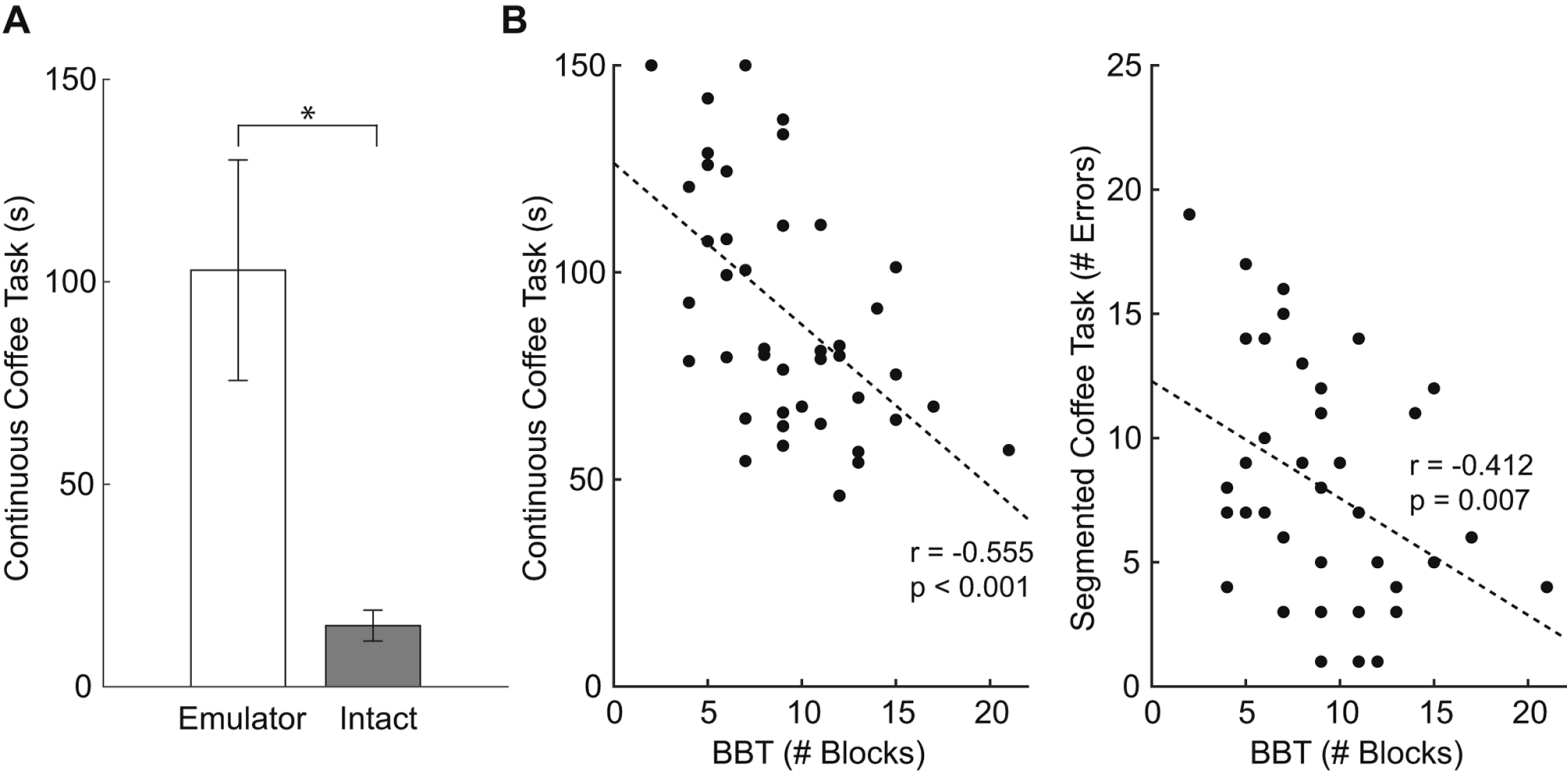
(**A**) Comparison of completion time for the Continuous Coffee Task between trials completed with the prosthetic emulator emulatator and those completed with intact hands were quantified as a measure of known-group validity. (**B**) Correlations between the outcomes of the Continuous and Segmented Coffee Tasks vs. outcomes of BBT were quantified as a measure of convergent validity

Participants completed the Continuous Coffee Task significantly faster with their intact limbs than when using the prosthetic emulator (*p* < 0.001; Fig. 4A). There were significant negative correlations between the BBT and Continuous Coffee Task (*r* = -0.555; *p* < 0.001; Fig. 4B) and Segmented Coffee Task (*r* = -0.412; *p* = 0.007) outcomes.

### Responsiveness

**Fig. 5:**
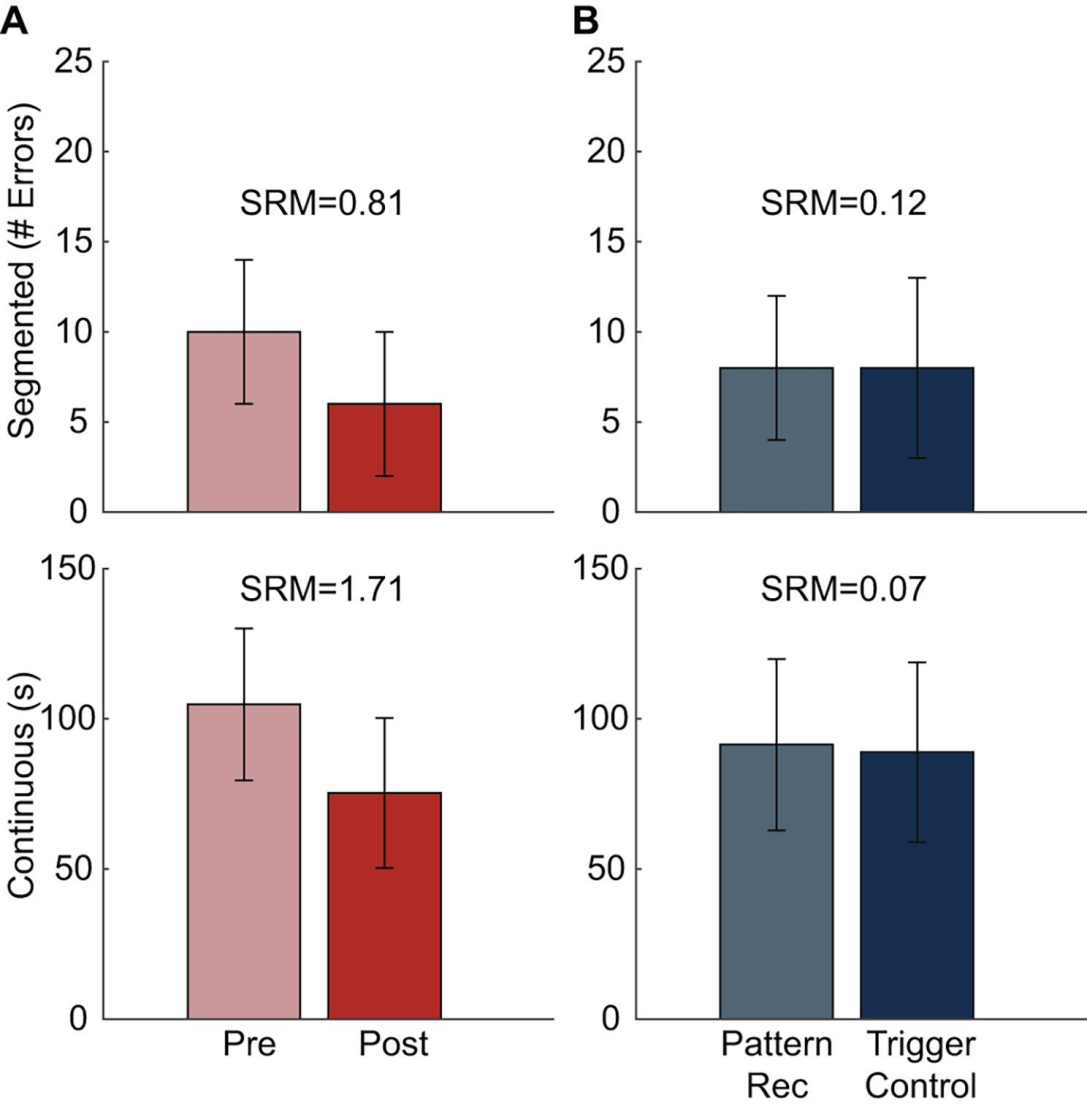
Responsiveness to change. (**A**) Average outcomes for the Segmented and Continuous Coffee Tasks Pre and Post-practice. (**B**) Average outcomes for the Segmented and Continuous Coffee Tasks with pattern recognition and trigger control. The standardized response means (SRM) is shown for each comparison. SRMs < 0.5 are interpreted as being not responsive to change

Both versions of the Coffee Task were responsive to practice (SRM = 1.71 and 0.81 for time and error, respectively; Fig. 5) with large effect sizes (d = 1.2 and 0.98 for time and errors, respectively). In contrast, neither task was responsive to control approach (SRM < 0.12) and both had small effect sizes (d < 0.13).

### Perceived workload

The average NASA-TLX perceived workload was 43.6 (range: 9.67–71.33), indicative of a somewhat high workload. No participants rated their workload as ‘very high.’

## Discussion

We developed two versions of a novel assessment, the Coffee Task, that quantifies prosthetic grip selection performance. Both the Segmented and Continuous Coffee Tasks had reasonable inter-rater reliability for clinical measures, known-group validity, and convergent validity. Despite the multiple steps needed to complete the task, it was feasible to learn and complete within a few minutes of instruction. No participants reported a very high workload for this task, which is particularly meaningful as the participants had no prior prosthetic experience. Accordingly, we expect the task to be easy to implement with regular users of multi-grip prostheses.

Neither version of the Coffee Task indicated significant ceiling or floor effects when completed by participants who were novel users of the prosthesis. As such, we may expect prosthesis users with more experience to perform with faster completion time during the Continuous Coffee Task and fewer or no errors during the Segmented Coffee Task. In these cases, administering both versions may be beneficial as the completion time could further differentiate skill level among participants who can complete the Segmented Coffee Task without errors. This is supported by the distribution of times for intact limbs (Fig. 3A). Likewise, we expect that prosthesis users with very low proficiency would still select an appropriate grip at least once. For instance, participants who completed the Continuous Coffee Task with maximum allowable time completed the Segmented Coffee Task with fewer than 25 errors. Together, we expect the Segmented Coffee Task is particularly useful in differentiating individuals with lower skill, while the Continuous Coffee Task is useful in differentiating individuals with more proficiency.

Both versions of the Coffee Task were significantly correlated with the BBT. The correlations were negative, indicating that those who moved more blocks during the BBT, also completed the Continuous Coffee Task faster and the Segmented Coffee Task with fewer errors. The moderate strength of the correlations (|r| > 0.412) between the Coffee Task and BBT suggest that the Coffee Task measures similar, but not redundant, aspect of manual dexterity as the BBT. This is expected given the design of the two tasks, as the BBT can be completed without an explicitly assigned prosthetic grip, while the Coffee Task was specifically constructed to assess performance during grip selection.

Both versions of the Coffee Task were responsive to training, although neither was responsive to changes in control approach. Therefore, the Coffee Task could be used to differentiate novel and experienced prosthesis users and track the progress of training over time. While the Coffee Task was unable to differentiate performance with trigger control and pattern recognition, it is unclear if these approaches would necessarily be differentiable in this study. Some studies postulate that pattern recognition is more intuitive than trigger control [14, 37] by eliminating the need for unnatural muscle contraction. However, successful use of pattern recognition relies on consistent application of EMG patterns collected through multiple socket-mounted electrodes. As such, some prosthesis users have expressed concerns regarding the extensive amount of training required to use pattern recognition reliably [7]. Given these considerations, it is likely that participants in this study varied how they acquired skills necessary to use the two control approaches, leading to a range of performance during the Coffee Task that averaged to a lack of responsiveness. While the Coffee Task was not responsive to change between trigger control and pattern recognition, it is possible that it may be able to differentiate performance between similar control approaches with different signal inputs. In support of this idea, a previous study that compared two pattern recognition controllers with different input signals (surface vs. intramuscular EMG) in a single prosthesis user with extensive experience with pattern recognition demonstrated longer completion times and more errors with surface EMG control during the Coffee Task [38].

There are several limitations to consider when interpreting results from this study. First, the study participants were healthy individuals who were using a myoelectric emulator. With the difference in musculature, myoelectric control of healthy individuals is not necessarily translatable to expected control in individuals with limb loss [39, 40]. It is also possible that some participants experienced fatigue that may have affected their performance. To limit this, we placed intentional breaks during the 30-minute practice and allowed participants to take breaks as necessary during Pre- and Post-, however it is possible that this break was insufficient. An additional limitation in the current construction of the Coffee Task is that it was limited to assessment of individuals using a right-handed prosthesis. To assess performance in individuals with left limb loss, the placements of the objects would be shifted such that the objects ipsilateral to the side of limb loss would align with the right-handed version of the task. While the expected outcomes of the assessment should not be influenced by the side of amputation, future development may consider obtaining validity measures during this variation of the task.

## Conclusions

Together, our findings suggest that the Coffee Task is an easily implementable valid and reliable assessment of multi-grip prosthetic performance. The Coffee Task was responsive to training, and thus can be used to track rehabilitation progress. Future work will establish the feasibility and reliability of this assessment in individuals with upper limb loss. The availability of an assessment tool that quantifies prosthetic grip selection performance will be imperative in understanding the functional benefits of multi-grip hands and designing hands that improve function in individuals with limb loss.

### Electronic supplementary material

Below is the link to the electronic supplementary material.

**Additional file 1:** is an error type classification rubric during the Segmented Coffee Task for trigger control

**Additional file 2:** is an error type classification rubric during the Segmented Coffee Task for pattern recognition

**Additional file 3:** is a figure illustrating trial-by-trial distribution of Coffee Task outcomes for the two control approaches in different time points (Pre/Post)

**Additional file 4:** is a figure illustrating error type classification count during the Segmented Coffee Task from Raters 1 and 2

## Data Availability

The datasets used and/or analyzed during the current study are available from the corresponding author on reasonable request.

## Abbreviations

ACMC: Assessment of Capacity for Myoelectric Control
AMULA: Activities Measure for Upper Limb Amputees
ANOVA: Analysis of Variance
BBT: Box and Block Test
EMG: Electromyography
ICC: Intra-Class Coefficient
LDA: Linear Discriminant Analysis
SHAP: Southampton Hand Assessment Procedure
SRM: Standard Response Mean
