# Supplementary material for: Development and validation of the coffee task: a novel functional assessment for prosthetic grip selection
Source: J Neuroeng Rehabil. 2024 Feb 8;21:21. doi: 10.1186/s12984-024-01307-y (PMC10851532; doi:10.1186/s12984-024-01307-y)
Supplement: Supplementary file 1 — Additional file 1: is an error type classification rubric during the Segmented Coffee Task for trigger control [file 12984_2024_1307_MOESM1_ESM.docx]

**Additional File 1**. Error type classification rubric for trigger control.

| **Error Type** | **1**  **Grasp Error** | **2**  **No Response**  **Error** | **3**  **User**  **Error** | **4.1**  **User**  **&**  **Software**  **Error** | **4.2**  **User**  **&**  **Software**  **Error** | **5**  **Mid-attempt Hand Open Error** | **6**  **Mid-attempt Grasp Error** | **7**  **Non-Transition Error** |
| --- | --- | --- | --- | --- | --- | --- | --- | --- |
| **What is the intended action?** | Transition to the designated grasp | Transition to the designated grasp | Transition to the designated grasp | Transition to the designated grasp | Transition to the designated grasp | Nothing | Nothing | Dropping objects without opening the hand |
| **How does the user attempt this intended action?** | Apply trigger for the designated grasp | Apply trigger for the designated grasp | Apply trigger for a different grasp | Apply trigger for a different grasp | Apply trigger for a different grasp | Nothing | Nothing |  |
| **What does the hand do?** | Transition to unintended grasp | No grasp transition | Transition to unintended grasp, correct trigger pairing | Transition to the designated grasp, incorrect trigger pairing | Open hand, transition to unintended grasp, incorrect trigger pairing | Open hand, fluttering | Transition to unintended grasp |  |
