# Supplementary material for: Development and validation of the coffee task: a novel functional assessment for prosthetic grip selection
Source: J Neuroeng Rehabil. 2024 Feb 8;21:21. doi: 10.1186/s12984-024-01307-y (PMC10851532; doi:10.1186/s12984-024-01307-y)
Supplement: Supplementary file 2 — Additional file 2: is an error type classification rubric during the Segmented Coffee Task for pattern recognition [file 12984_2024_1307_MOESM2_ESM.docx]

**Additional File 2**. Error type classification rubric for pattern recognition.

| **Error Type** | **1**  **Commanded decode classification error** | **2**  **Commanded software error (no response)** | **3**  **Un-commanded mid-attempt grasp selection** | **4**  **Un-commanded mid-attempt hand open** | **5**  **Non-Transition Error** |
| --- | --- | --- | --- | --- | --- |
| **What is the intended action?** | Transition to the designated grasp | Transition to the designated grasp | Nothing | Nothing | Dropping objects without opening the hand |
| **What does the hand do?** | Transition to an unintended grasp | No grasp transition | Transition to an unintended grasp | Open hand, fluttering |  |
