# Supplementary material for: Development and validation of the coffee task: a novel functional assessment for prosthetic grip selection
Source: J Neuroeng Rehabil. 2024 Feb 8;21:21. doi: 10.1186/s12984-024-01307-y (PMC10851532; doi:10.1186/s12984-024-01307-y)

**Additional File 3**. Trial-by-trial distribution of outcomes for each condition (Pattern recognition – PR; Trigger control – TC). There were no significant effects of trial number on outcomes.


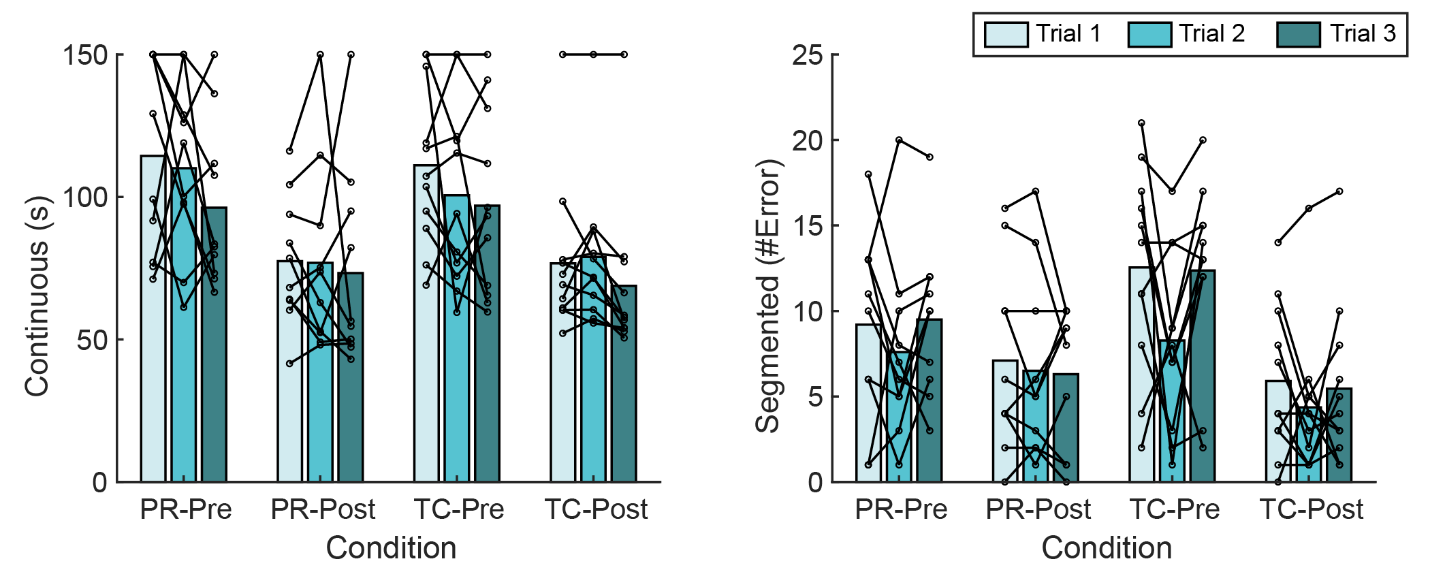

Supplement: Supplementary file 3 — Additional file 3: is a figure illustrating trial-by-trial distribution of Coffee Task outcomes for the two control approaches in different time points (Pre/Post) [file 12984_2024_1307_MOESM3_ESM.docx]
