# Supplementary figures and images for: Development and validation of the coffee task: a novel functional assessment for prosthetic grip selection
Source: J Neuroeng Rehabil. 2024 Feb 8;21:21. doi: 10.1186/s12984-024-01307-y (PMC10851532; doi:10.1186/s12984-024-01307-y)

**Additional File 4**. Error type classification count for Rater 1 and Rater 2.


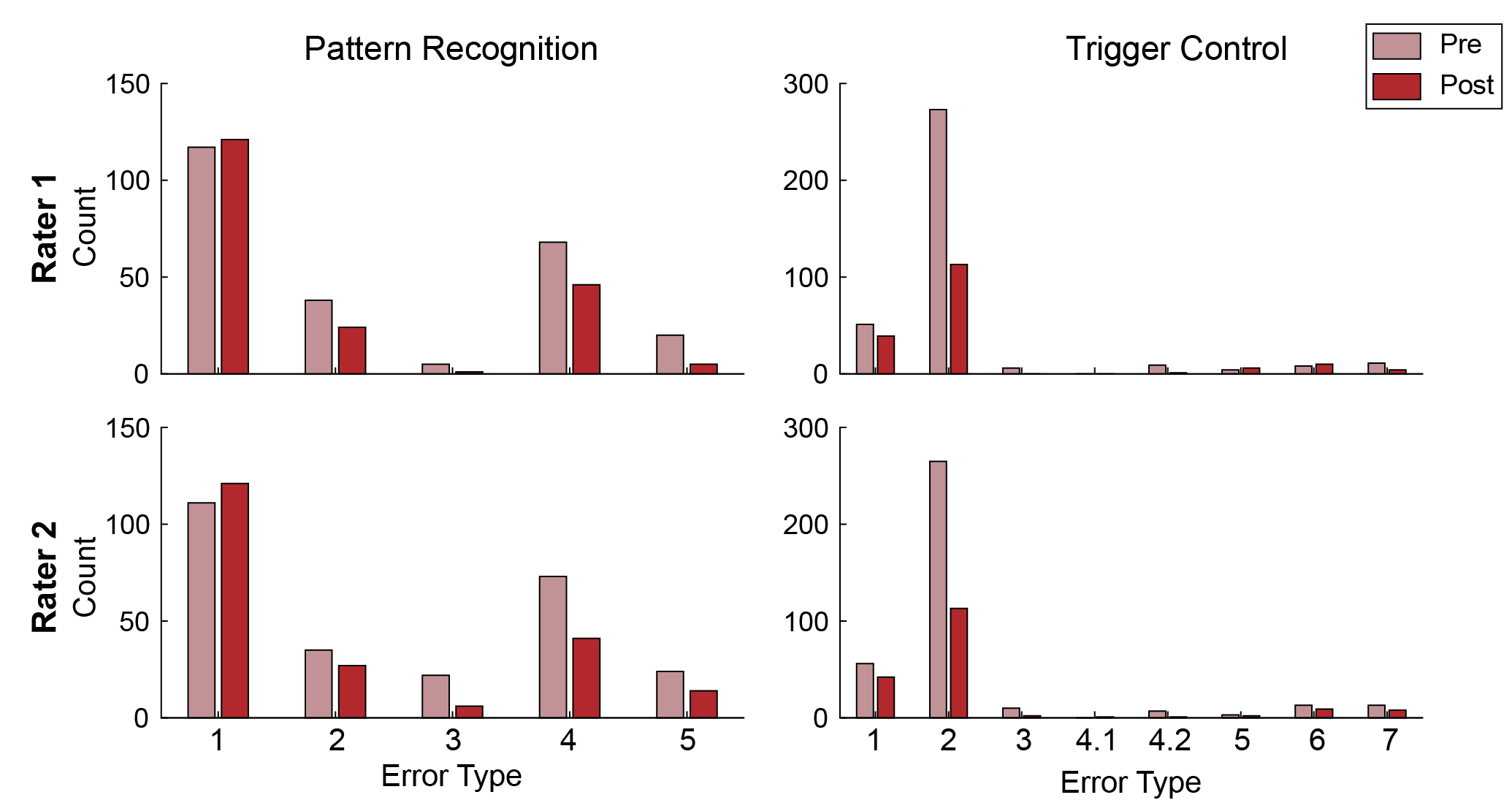

Supplement: Supplementary file 4 — Additional file 4: is a figure illustrating error type classification count during the Segmented Coffee Task from Raters 1 and 2 [file 12984_2024_1307_MOESM4_ESM.docx]
